# Brain pathology in first-episode psychosis: Magnetization transfer imaging provides additional information to MRI measurements of volume loss

**DOI:** 10.1016/j.neuroimage.2009.07.037

**Published:** 2010-01-01

**Authors:** Gary Price, Mara Cercignani, Elvina M. Chu, Thomas R.E. Barnes, Gareth J. Barker, Eileen M. Joyce, Maria A. Ron

**Affiliations:** a Institute of Neurology, University College London, Queen Square, London WC1N 3BG, UK; b Imperial College Faculty of Medicine, Charing Cross Campus, London, UK; c King's College London, Institute of Psychiatry, Department of Clinical Neuroscience, Centre for Neuroimaging Sciences, London, UK; d Neuroimaging Laboratory, Fondazione Santa Lucia, IRCCS, Rome, Italy

## Abstract

**Background:**

Loss of brain volume in first-episode psychosis can be detected using conventional magnetic resonance imaging (MRI), but subtle changes – not leading to reduction in volume – that may contribute to clinical and cognitive abnormalities, may go undetected. Magnetization transfer imaging (MTI), a technique more sensitive to subtle neuropathological changes than conventional MRI, could yield important information on the extent and nature of structural abnormalities.

**Methods:**

Forty-eight patients (33 males) from a population-based sample with first-episode psychosis (41 with schizophrenia and 7 with schizoaffective psychosis) and 47 healthy volunteers (27 males) were studied. Differences in magnetization transfer ratio (MTR) and white and grey matter volumes between groups were investigated.

**Results:**

In patients, MTR was reduced in right entorhinal cortex, fusiform, dentate and superior frontal gyri and in left superior frontal and inferior/rostral cingulate gyri. Grey matter volume was reduced in right insula, frontal operculum and middle and superior temporal gyri and in left middle temporal gyrus. Grey matter volume increases were seen in patients in the superior frontal gyrus. White matter volume loss was found adjacent to grey matter loss. In patients MTR was lower in all areas of volumetric differences between groups suggesting that both changes may be related. Similar findings were observed when patients with schizoaffective psychosis were removed from the analysis. The correlations between clinical and MRI parameters did not survive correction for multiple comparisons.

**Conclusions:**

MTI frontal and temporal abnormalities suggesting neuroaxonal and myelin changes were more extensive in our patients than those detected with conventional MRI. Our findings also suggest that there is regional variation in the severity of structural brain abnormalities.

## Introduction

Brain imaging abnormalities are present in the first episode of schizophrenia (Borgwardt et al., 2008; Steen et al., 2006) and its prodrome (Lappin et al., 2007; Pantelis et al., 2003; Borgwardt et al., 2006). Predominantly frontal grey matter losses, but also extending to temporal and limbic regions at psychosis onset, (Fornito et al., 2009; Glahn et al., 2008; Lui et al., 2009; Sun et al., 2009) with further losses later in the illness, have been reported, (Lieberman et al., 2001; Mathalon et al., 2001; Rapoport et al., 1997; DeLisi et al., 1997) although there is no evidence to suggest neurodegeneration and a variety of environmental factors may account for these findings (Rais et al., 2008). Magnetization transfer imaging (MTI), an MRI modality capable of detecting structural changes in the absence of volumetric loss, may prove helpful in studying the subtle abnormalities present in first-episode psychosis and their natural history. MTI (Wolff and Balaban, 1989) is based on the interaction between protons bound to macromolecules (e.g. myelin and cell membranes) and protons in free water. An off-resonance pre-pulse allows the magnetization of bound protons to be saturated and through chemical exchange and cross-relaxation, magnetization is transferred to the free pool of protons, reducing the MR signal. The degree of signal loss is expressed as the magnetization transfer ratio (MTR), a highly reproducible measure in normal subjects (Barker et al., 1996). MTR depends on the density of macromolecules, and pathological processes that damage the macromolecular structure result in MTR reductions, often in the absence of demonstrable atrophy in neurological illness such as multiple sclerosis (Audoin et al., 2006; Fernando et al., 2005) and epilepsy (Flugel et al., 2006; Rugg-Gunn et al., 2003).

We have previously reported bilateral MTR reductions in the medial prefrontal cortex, insula and adjacent white matter in patients with first-episode psychosis without volumetric loss (Bagary et al., 2003). The study we present here was conducted in a larger, unrelated group of patients with first-episode psychosis using an improved, three-dimensional (3D)-MTI sequence with much thinner slices than our previous studies, along with voxel-based morphometry (VBM) using a matched 3D (volumetric) acquisition. We aimed (i) to see if similar MTI abnormalities could be detected in a larger sample of patients using optimized MTI methodology, and (ii) to elucidate the link between MTR and volumetric changes, and to explore MRI/clinical correlations.

## Methods

The patients were recruited as part of a prospective, longitudinal study of first-episode psychosis in West London. Patients eligible for the study were screened using the WHO Psychosis Screen (Jablensky et al., 1992). Patients were initially included if aged between 16 and 50 years, presenting with a psychotic illness for the first time and had received no more than 12 weeks of antipsychotic medication. In addition to the WHO Psychosis Screen (Jablensky et al., 1992), clinical notes were reviewed to exclude a previous first episode of psychosis. Clinical ratings were performed within 12 weeks of commencing antipsychotic medication. Diagnoses was ascertained using a structured interview, the diagnostic module of the Diagnostic Interview for Psychosis (DIP, Jablensky et al., 2000). Patients and their community mental health teams were contacted 1 year later at which time the diagnosis was reviewed by two psychiatrists (TREB and EMJ). Not all patients that were eligible underwent MRI scanning; about half of those approached did not participate with scanning either because they refused or were unable to tolerate the scanning procedure. Forty-eight patients (33 males, 15 females) were included in the study. Forty-one had a final DSMIV diagnosis of schizophrenia and seven of schizoaffective disorder.

Forty-seven healthy subjects (27 males and 20 females) served as control subjects and were recruited from the same catchment area as patients by advertising in local colleges and hospitals. For the controls, participants were excluded if they had a history of psychiatric illness in themselves or their first-degree relatives. For both groups, exclusion criteria were previous head injury or other neurological illness or endocrine disorder affecting brain function, such as epilepsy and thyroid disease, and drug or alcohol dependence.

Symptom severity and range were assessed with the Scales for the Assessment of Positive (Andreasen, 1984a,b) and Negative (Andreasen, 1984a,b) Symptoms, the Young Mania Scale (Young et al., 1978) and the Hamilton Rating Scale for Depression (Hamilton, 1960). Age of onset and duration of untreated psychosis were established using the Symptom Onset in Schizophrenia inventory (Perkins et al., 2000). The Alcohol and Drug Use Scales (Drake et al., 1990) were used to determine dependency. Handedness was assessed using the Annett scale (Annett, 1970).

Permission for the study was obtained from Merton, Sutton and Wandsworth, Riverside and Ealing research ethics committees. All participants gave written informed consent and received an honorarium for taking part in the study.

### MRI data acquisition

MRI was performed with a GE Signa 1.5 T scanner (General Electric, Milwaukee, WI, USA). A T_1_-weighted volumetric image was obtained using a 3D inversion recovery prepared spoiled gradient-recalled echo (SPGR) sequence in the axial plane. This sequence obtained 156 contiguous axial slices (TE = 5 ms, TR = 14 ms, matrix = 256 × 128, field of view (FOV) = 31 × 16 cm^2^, slice thickness = 1.2 mm, NEX = 1, excitation flip angle 20°, bandwidth 15.63, inversion time 450 ms). In addition, a 3D MT-prepared SPGR was performed (TE = 5 ms, TR = 22 ms, NEX = 1, flip angle = 10°, bandwidth 15.63 kHz, with 350°, 6.4 ms, Hamming-apodized three-lobe sinc pulse applied 2 kHz from water resonance for MT saturation), with slice thickness, matrix size and FOV matched to the inversion recovery prepared SPGR. The MT sequence generated two volumes, one with and one without MT saturation.

### Image processing

Image processing was performed on a Sun workstation (Sun Microsystems, Santa Clara, CA, USA). Initially the two volumes from the MT SPGR sequence were co-registered to each other with an affine transformation using FLIRT (Jenkinson et al., 2002), to compensate for involuntary movement during acquisition. MTR maps were then calculated on a pixel-by-pixel basis using the formula MTR = ([Mo − Ms] / Mo) × 100 percent units (pu), where Ms and Mo are mean signal intensities with and without the saturation pulse, respectively.

Further processing was then performed using SPM2 (Wellcome Department of Cognitive Neurology, London, UK) in MATLAB (MathWorks, Natick, MA, USA) with an “optimized VBM” approach (Good et al., 2001) and matched steps on the MTR maps:

#### Co-registration of MTR and T_1_-weighted images

Non-MT-weighted scans from the MT sequence were co-registered to the corresponding T_1_-weighted volume; only a rigid body (6-degree-of-freedom) transformation was necessary for this stage, as the scans both used high resolution 3D sequences with very similar acquisition parameters and the aim was simply to correct for intra-subject, inter-scan, head motion. The same transformation was applied to the MTR maps. All images were thus in the space of the T_1_-weighted volume (Studholme et al., 1997).

#### Segmentation of the T_1_-weighted images in native space

This was performed in native space using the SPM2 Bayesian algorithm (Ashburner and Friston, 1997) to assign voxels as grey matter, white matter, or cerebrospinal fluid.

#### Normalization

Grey matter images were spatially normalized into MNI (Montreal Neurological Institute) stereotactic space using the SPM2 *a priori* grey matter image as a template. Normalization was first obtained using a 12-parameter affine transformation and was then optimized using 16 non-linear warps (Ashburner and Friston, 1997). The optimized transformation parameters were then applied to the original T_1_-weighted images and MTR maps.

#### Segmentation

Normalized T_1_-weighted images were segmented to produce grey matter, white matter, and cerebrospinal fluid segments. This iterative procedure was used because normalization is ideally performed on segmented images, while segmentation is better performed in stereotactic space (Good et al., 2001).

#### Modulation

Possible changes in regional shape to fit the template during warping affects the quantification of grey and white matter density, resulting in an artificially altered tissue density. The true amount of tissue volume per voxel is retrieved by dividing the image intensity by the regional volumetric deformation factor—a procedure referred to as Jacobian modulation (Ashburner and Friston, 2001).

#### Smoothing

MTR maps and segmented images of grey and white matter were smoothed with a 12 mm FWHM Gaussian kernel to address possible residual registration errors and inter-individual variability and to ensure the normality requirements of parametric statistics were met. As the size of the kernel can affect the results of the analysis, (Jones et al., 2005; Honea et al., 2005) we selected a 12 mm kernel that is often used in schizophrenia studies (McClure et al., 2006a,b; Spencer et al., 2007; Yamada et al., 2007) and is less likely than smaller kernels to detect group differences of uncertain significance.

#### Volumetric and MTR group comparisons

These were made using SPM2 in the framework of the general linear model, including the total grey or white matter volume as a covariate for the volumetric data, and total brain volume for the whole-brain MTR analysis. Differences in these measures in patients relative to controls were investigated using *t* tests and corrected for multiple comparisons using a family-wise error correction at *P* < 0.05. These *t* tests produced statistical parametric maps depicting regions that differed significantly between groups.

#### Post-hoc clinical/MRI correlations

Means for MTR and for grey and white matter volumes were calculated for each cluster where differences between groups were present, using the “volume-of-interest” option in SPM2 and correlations with positive, negative and disorganization symptoms explored using Pearson's correlations.

#### Post-hoc MTR group comparisons in areas of volume change

To explore the relationship between MTR and volume changes, the mean MTR values of patients and controls were compared for the brain regions where grey or white matter volumes differed significantly. An image mask for these regions was constructed and overlaid on each subject's smoothed whole-brain MTR images. Mean MTR values for the areas covered by this mask were obtained for each subject, and group comparisons made using an independent-samples *t* test.

## Results

The mean age for patients was 26.2 years (range 16–50) and for controls, 24.8 years (range 16–37). Four patients and six controls were left-handed. No subject fulfilled criteria for alcohol or drug dependency.

All patients received antipsychotic medication (23 olanzapine, 16 risperidone, 4 aripiprazole, 3 amisulpride, 1 quetiapine, 1 haloperidol) and 7 were also prescribed antidepressants. The average duration of treatment before scanning was 77 (9–186) days. For those taking olanzapine, the average dose was 14.25 mg and average duration of treatment before scanning was 83 days. For those taking risperidone, the average dose was 3.5 mg with an average duration of treatment prior to scanning of 79 days.

Those patients that were scanned did not significantly differ from those that refused or were unable to be scanned in terms of total SAPS scores (scanned mean = 30.8, unscanned mean = 30.4; *t* = 0.123, df = 119, *P* = 0.903) and total SANS scores (scanned mean = 16.3, unscanned mean = 18.8; *t* = 0.426, df = 119, *P* = 0.671). There were also no significant differences in terms of duration of untreated illness (scanned mean = 33 months, unscanned mean = 27 months; *t* = 1.049, df = 119, *P* = 0.296).

There were no significant differences between patients and controls in age (*t* = − 0.043, df = 93, *P* = 0.348), gender (*χ*^2^ = 1.304, df = 1, *P* = 0.254) or handedness (*χ*^2^ = 0.495, df = 1, *P* = 0.482). Current IQ data were not available for all subjects and more subjects participated with premorbid IQ estimation (Wechsler Test of Adult Reading, Pearson, TX, USA) than current IQ testing. For those subjects that had premorbid IQ estimates available, patients (*n* = 30, mean predicted IQ = 95, SD 14.2) had significantly lower estimates than controls (*n* = 36, mean predicted IQ = 103, SD 10.1); (*t* = 2.674, df = 64, *P* = 0.01).

### MTR group differences

In patients, MTR reduction in the right hemisphere was observed in the fusiform gyrus, entorhinal cortex, dentate gyrus, and superior frontal gyrus. Areas of MTR reduction in the left hemisphere were seen in the superior frontal gyrus and inferior/rostral cingulate gyrus. There were no MTR increases in patients relative to controls.

### MRI volumetric group differences

#### Grey matter

In the patient group, grey matter loss was seen in the middle temporal gyrus, insula, frontal operculum and superior temporal gyrus on the right and in the left middle temporal gyrus.

A bilateral increase in grey matter volume involving the superior frontal gyrus and a small region of the right superior temporal gyrus was present in patients compared with controls.

#### White matter

White matter loss occurred in patients in areas bordering the right insular gyrus and the middle and superior temporal gyri and in the left temporal subgyral and insular regions. There were no areas of increased white matter volume in patients.

**Fig. 1:**
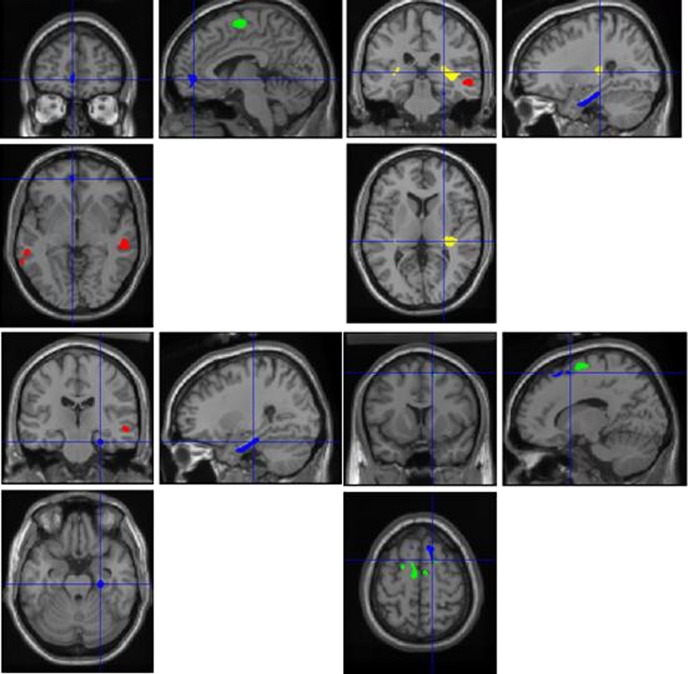
Statistical parametric maps depicting the different regions where MTR and brain volume differed significantly between groups (*P* < 0.05, family-wise error corrected for multiple comparisons), overlaid on a standard template using SPM2. Blue denotes MTR reduction; red, grey matter volume loss; and yellow, white matter reductions (patient group). Green denotes grey matter volume increases in the patient group.

**Fig. 2:**
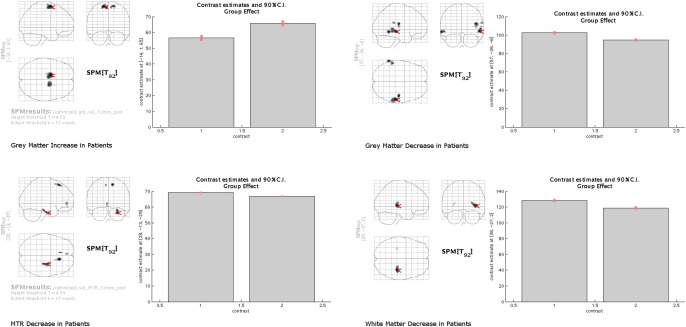
90% Confidence Intervals in the most significant clusters for white and grey matter decreases in patients, grey matter increases in patients and MTR reductions in patients. Control (*n* = 47) data are plotted first (1 = Controls, 2 = Patients) and coordinates are shown in the glass brain (arrow) and vertical axis.

Cluster sizes and coordinates are shown in Table 1 and Fig. 1 and plots for 90% Confidence Intervals for the most significant cluster in each group difference are shown in Fig. 2.

### Analysis excluding schizoaffective patients

**Fig. 3:**
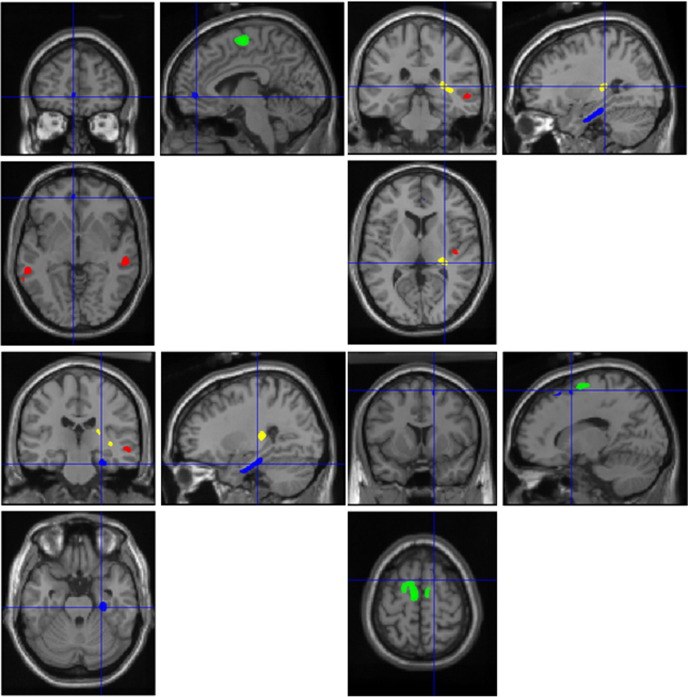
Statistical parametric maps of schizophrenia only patients compared to controls. The maps depict the different regions where MTR and brain volume differed significantly between groups (*P* < 0.05, family-wise error corrected for multiple comparisons), overlaid on a standard template using SPM2. Blue denotes MTR reduction; red, grey matter volume loss; and yellow, white matter reductions (patient group). Green denotes grey matter volume increases in the patient group.

When the 7 patients with schizoaffective psychosis were excluded from the analysis (*n* = 41) very similar findings in terms cluster size and distribution for volume loss, grey matter volume increases in patients and MTR differences were revealed. This distribution is shown in Fig. 3.

### Post-hoc clinical/MTR and volumetric correlations

Severity of total positive symptoms score (SAPS) and the severity of the flattening of affect subscale score from the SANS correlated with MTR reduction in the right medial temporal region (coordinates 28 − 14 − 24) (Pearson's correlation coefficients − 0.285, *P* = 0.05, and − 0.313, *P* = 0.03, respectively). Flattening of affect correlated with MTR reduction in the left fronto/cingulate region (Pearson's correlation coefficient − 0.296, *P* = 0.041), and longer duration of untreated illness with MTR reduction in the right superior frontal gyrus (coordinates 13, 27, 52) (Pearson's correlation coefficient − 0.353, *P* = 0.014). These correlations were no longer significant after Bonferroni corrections for multiple comparisons were applied. There were no significant correlations between clinical ratings and grey or white matter volume loss. There were also no significant Pearson's correlations for risperidone or olanzapine dose × duration in areas of grey and white matter loss and MTR reductions in patients, as well as grey matter increase in patients.

### Post-hoc group comparisons of MTR in areas of volume change

In areas where the grey matter volume was decreased in patients, mean MTR values were 35.47 pu (SD = 1.15) for patients and 36.11 pu (SD = 0.91) for controls (*t* = − 2.97, df = 93, *P* = 0.004). In areas where white matter volume was decreased in patients, mean MTR values were 42.06 pu (SD = 1.10) for patients and 42.67 pu (SD = 0.97) for controls (*t* = − 2.89, df = 93, *P* = 0.005). In areas where the grey matter volume was increased in patients, mean MTR values were 32.49 pu (SD = 1.45) for patients and 33.37 pu (SD = 1.49) for controls (*t* = − 2.93, df = 93, *P* = 0.004).

## Discussion

Using two complementary modalities of structural MRI, we have obtained results suggesting that frontal and temporal abnormalities are present in patients with first-episode psychosis. The severity of these abnormalities was greater in temporal regions, and more so in the right hemisphere. Our results also suggest that MTI provides additional, complementary information to that of volumetric MRI regarding the extent of pathological changes early in the course of the illness.

Loss of grey matter in fronto-temporal regions has been reported in other studies of patients with first-episode psychosis, (Choi et al., 2005; Chua et al., 2007; Pantelis et al., 2003) although the fine localization of volumetric changes varies between studies, presumably because of the heterogeneity of patient populations and methodologies. Loss of fronto-temporal white matter has been reported less frequently in first-episode psychosis, (Ho et al., 2003; Whitford et al., 2007) although reduction in the numbers of oligodendrocytes, (Uranova et al., 2004) abnormal expression of myelin-related genes, (Hakak et al., 2001) and changes in the integrity of white matter tracts shown using diffusion-tensor MRI (Price et al., 2007, 2008) suggest that white matter abnormalities are also present early in the disease. Right-sided volumetric losses in temporal and frontal regions have also been reported in other studies using voxel-based morphometry, although less frequently than left-sided ones (Honea et al., 2005).

In post-mortem brains, imaging correlations suggest that white matter MTR depends on myelin and, to a lesser extent, on axonal density, (Schmierer et al., 2007) and MTR reduction is strongly associated with tissue damage. The pathological abnormalities leading to MTR changes in grey matter are less well documented, but with less abundant myelin, decrease in the number and/or size of neurones, loss of dendritic arborisation, and changes in proteins and phospholipids in cell membranes are likely to account for reductions in MTR. It is also unclear how tissue changes detected by MTR relate to any reductions in brain volume also seen. In a follow-up study of patients with multiple sclerosis, Khaleeli et al. (2007) reported only a moderate correlation between MTR decreases and grey and white matter atrophy over one year, although atrophy did not fully explain the MTR changes and the authors concluded that, although MTR measures need to be interpreted in the context of atrophy, MTR was also an independent marker of pathology. Thus, MTR appears to detect subtle neuropathological changes, not all of which result in detectable tissue volume loss. In our patients, MTR changes were more extensive than grey and white matter volumetric losses, particularly in frontal regions. This could indicate more severe abnormalities in some brain regions (i.e. the temporal as compared with the frontal lobes) and/or subtle differences in the nature of neuropathological changes. In our previous 3-year first-episode follow-up study of a different small group of patients we failed to detect MTR changes over time (Price et al., 2006) and a larger follow-up study would be needed to elucidate the relationship between changes in MTR and volumetric loss.

The lack of overlap between areas of volume loss and MTR reduction in the temporal lobe deserves comment. Differences in volumetric and MTR data analysis are unlikely to explain this dissociation, although grey and white matter data were modulated and therefore weighted for the effects of local atrophy, whereas MTR data were not. Because of the larger number of voxels, more stringent corrections for multiple comparisons were applied to the MTR than to the volumetric data as MTR analysis was performed over the whole brain, while grey matter volume was by definition assessed only over the grey matter; however lowering the acceptable level of significance did not change our findings, and further removes the possibility that the MTR results are artifactual. On the other hand, mean MTR was lower in the patient group in all areas of volume loss in post-hoc comparisons. This supports the findings of Khaleeli et al. (2007) suggesting that while there is a link between MTR reduction and loss of brain volume, MTR may be an independent marker of pathology. The possibility that MTR is more sensitive that volumetric loss to the current or cumulative effects of antipsychotic medication remains a possibility, however, the fact that a dissociation between these two parameters has also been described in other studies of patients not exposed to neuroleptics (Khaleeli et al., 2007) makes this unlikely.

The grey matter volume increase in the superior frontal gyrus, an area not generally associated with abnormalities in schizophrenia, was unexpected. Since MTR reduction was seen in this area in the patient group, it is possible that concomitant changes in SPGR signal intensity at the grey/white matter boundary may have resulted in tissue misclassification. An alternative explanation is that exposure to atypical antipsychotics (Lieberman et al., 2005) could have caused grey matter increases. Our findings of a focal increase in grey matter volume in the superior frontal gyri have support from other studies that have found grey matter volume increases in this region (McClure et al., 2006a,b) and other cortical frontal regions (Molina et al., 2005; Stip et al., 2009), although these and other studies (Garver et al., 2005) also show medication-related cortical thickening that tends to be more diffuse than the very focal change described here.

Correlations between symptom severity and volumetric loss in prefrontal and temporal regions have been reported in first-episode psychosis, (Kim et al., 2003) and others (Roth et al., 2004) have found predominantly right-sided fronto-temporal volumetric loss in those with high negative symptom scores. The reduction of MTR in fronto-temporal regions with increased symptom severity in our patients echoes previous reports, even if correction is modest. The loss of these correlations after multiple comparison adjustment is probably associated with medication-related symptomatic improvement. On the other hand our findings add support to those of Ho et al. (2003) who failed to find an association between duration of untreated psychosis and imaging abnormalities.

This is to date the largest MTI and volumetric study of first-episode psychosis. In our previous study, (Bagary et al., 2003) involving a different group of 30 first-episode patients and 30 controls, MTR reductions were present in the medial prefrontal cortex, insula, and fasciculus uncinatus without loss of brain volume. In the study reported here, the more sophisticated image acquisition and larger sample size are likely to account for the more extensive areas of MTR reduction and volumetric loss, as otherwise the characteristics of the subjects in the two studies were similar. In our previous MTI study of patients with chronic schizophrenia, (Foong et al., 2001) we reported more diffuse cortical abnormalities, prominent in the inferior and middle frontal, inferior and middle temporal, and superior occipital gyri, with extension into the white matter of the uncinate fasciculus. The findings of our two previous studies are not strictly comparable with those reported here, as there were differences in sample sizes, patient populations, MTI sequences and image analysis methods (SPM2 versus SPM99). However, all three studies revealed frontal and/or temporal MTR abnormalities with little or no loss of brain volume, although the fine localization of abnormalities differed between studies. Outside our group, others have reported small MTR reductions in patients with chronic schizophrenia in periventricular regions and temporal lobes (Antosik-Biernacka et al., 2006) or more significant in fronto-occipital and fronto-thalamic connections (Kubicki et al., 2005).

The use of a 3D MTI sequence, with high spatial resolution, matched to that of the volumetric scan, should improve the accuracy in the localization of abnormal areas. Nevertheless, the smoothing process, needed to render the residuals of the model fitted to the data more normally distributed, is likely to introduce some degree of partial volume effect. The size of the smoothing filter is known to affect the results (Jones et al., 2005) and we cannot exclude the possibility of finding a slightly different pattern of abnormalities with a smaller smoothing kernel.

The exclusion of the small number of patients with schizoaffective illness in our sample did not alter the main findings. The small number of schizoaffective patients did not allow us to examine their imaging in detail but there is evidence that volumetric loss in the neocortex (Nakamura et al., 2007) and prefrontal MTR reductions are also present in affective psychosis (Bruno et al., 2004).

The results reported here extend those of our previous studies and go some way towards elucidating the link between MTR and volumetric loss. They also provide evidence that MTI can detect structural brain changes not visible with conventional MRI and support the view that the combined use of these two MRI modalities may prove useful in elucidating structural brain abnormalities in psychosis and in determining their natural history.

## Figures and Tables

**Table 1:** Number of voxels and Talairach coordinates of significant clusters (*P* < 0.05, corrected for family-wise error) in SPM maps showing differences between patients and control subjects.

Change in patients	Number of voxels in cluster^a^	Max Talairach coordinates (*x y z*)	Area
MTR decrease	1417	28 − 14 − 24	Right. *Grey matter*: dentate gyrus, fusiform, uncus, entorhinal cortex. *White matter*: periventricular around temporal horn
439	− 4 48 − 3	Left. *Grey matter*: inferior and superior rostral gyrus, cingulate, gyrus rectus and underlying *white matter*
775	13 27 52	Right. *Grey matter*: superior frontal gyrus and underlying *white matter*
187	− 8 32 52	Left. *Grey matter*: superior frontal gyrus
Grey matter decrease	710	− 64 − 48 − 3	Left. Middle temporal gyrus
1566	56 −25 −4	Right. Middle temporal gyrus
577	45 − 16 18	Right. *Grey matter*: frontal operculum/insular gyrus
281	64 − 32 22	Right. *Grey matter*: superior temporal gyrus/planum temporale/insula
Grey matter increase	65	18 13 52	Right. Superior temporal gyrus
3274	− 14 3 61	Left. Superior frontal gyrus
1728	18 − 5 67	Right. Superior frontal gyrus
White matter decrease	2045	38 − 26 3	Right. Posterior internal capsule. White matter adjacent to insular gyrus, putamen and superior temporal gyrus
74	− 29 − 27 11	Left. Adjacent to insular gyrus

MTR, magnetization transfer ratio.

a < 10 voxelss not reported.
